# Extending Protein Domain Boundary Predictors to Detect Discontinuous Domains

**DOI:** 10.1371/journal.pone.0141541

**Published:** 2015-10-26

**Authors:** Zhidong Xue, Richard Jang, Brandon Govindarajoo, Yichu Huang, Yan Wang

**Affiliations:** 1 School of Software Engineering, Huazhong University of Science and Technology, Wuhan, Hubei, 430074, China; 2 Department of Computational Medicine and Bioinformatics, University of Michigan, Ann Arbor, MI, 48109, United States of America; 3 School of Life Science and Technology, Huazhong University of Science and Technology, Wuhan, Hubei, 430074, China; NCBS-TIFR, INDIA

## Abstract

A variety of protein domain predictors were developed to predict protein domain boundaries in recent years, but most of them cannot predict discontinuous domains. Considering nearly 40% of multidomain proteins contain one or more discontinuous domains, we have developed DomEx to enable domain boundary predictors to detect discontinuous domains by assembling the continuous domain segments. Discontinuous domains are predicted by matching the sequence profile of concatenated continuous domain segments with the profiles from a single-domain library derived from SCOP and CATH, and Pfam. Then the matches are filtered by similarity to library templates, a symmetric index score and a profile-profile alignment score. DomEx recalled 32.3% discontinuous domains with 86.5% precision when tested on 97 non-homologous protein chains containing 58 continuous and 99 discontinuous domains, in which the predicted domain segments are within ±20 residues of the boundary definitions in CATH 3.5. Compared with our recently developed predictor, ThreaDom, which is the state-of-the-art tool to detect discontinuous-domains, DomEx recalled 26.7% discontinuous domains with 72.7% precision in a benchmark with 29 discontinuous-domain chains, where ThreaDom failed to predict any discontinuous domains. Furthermore, combined with ThreaDom, the method ranked number one among 10 predictors. The source code and datasets are available at https://github.com/xuezhidong/DomEx.

## Introduction

Proteins consist of one or several stable, compact, and autonomously folding substructures, which are referred to as domains. The identification of protein domains plays an important role in determining protein structures by experimental methods including Nuclear Magnetic Resonance (NMR) and X-ray crystallography[1,2]. Meanwhile, it is also a preliminary step in computational methods of protein structure prediction [3–5]. Moreover, detailed knowledge of domains is essential to advancing our understanding of protein function and evolution [6,7].

Although protein domains usually have a single continuous segment of protein chain, there are still many domains formed from two or more nonsequential segments, which are called “discontinuous domains”[8]. For example, 28, 279 out of the 181,356 domains (~15%) are discontinuous in the CATH3.5 library[9,10] and nearly 16,761 proteins (~18%) have at least one discontinuous domain based on the domain classifications by DomainParser2[11] in the PDB library.

Over the last three decades, a number of methods have been developed to identify protein domains, which are roughly classified into two categories according to their input data: structure or sequence. The structure-based methods can accurately identify continuous and discontinuous domains from the atomic coordinates of proteins [8,11–15]. The sequence-based methods predicting domains from sequences alone have obtained some progress in predicting continuous domains. Even including tertiary structure libraries like CATH[9,10], SCOP[16], SMART[17] that provide domain partitions of continuous and discontinuous domains, few sequence-based methods can predict the discontinuous domains. Then the discontinuous domain prediction is an open and challenging problem.

An accurate discontinuous domain prediction includes predicting the accurate domain boundaries and the number of segments within one discontinuous domain. Currently, the sequence-based methods mainly focused on domain number and boundary prediction. DGS[18] guesses the domain number and further infers domain boundaries by predicting the size and the segment number of domains. DomCut[19] predicts inter-domain linker regions based solely on amino acid sequence composition information. Pfam[20–22], EVEREST[23,24] ADDA[25], and FiefDom[26] focus on domain boundaries prediction based on homologous alignments. CHOPnet[27],Dompro[28], DomNet[29], PPRODO[30], DROP[31] and DOBO[32] use different machine learning methods to identify domain boundaries.

Some methods such as SnapDRAGON[33], RosettaDom[34] and OPUS-DOM[35] first constructed a 3D model and then extracted domain boundaries with structure-based domain partition tools such as DAIL[13], PDP[12] and DomainParser [11]. Although these methods can detect discontinuous domains, the success of the domain assignments relies on the correctness of the predicted models, which are applicable only to small proteins[4]. DomainDiscovery[36] was developed to predict discontinuous domains mainly based on the predicted inter-residue contact interaction values, while the accuracy of long-range contacts prediction from the sequence alone is very low[37]. ThreaDom[38] uses a template cluster method to detect discontinuous domain based on the meta-server threading program LOMETS[39]. However, it will fail to identify discontinuous domains if there is no available template deposited in the PDB. And it didn’t use the domain information from the sequence domain libraries, such as Pfam [20–22] et al.

In this work, we present a new strategy, DomEx, to enable continuous domain boundary predictors to predict discontinuous domains based on the sequence segment assembly. A template similarity score, symmetric index score and a profile-profile alignment score were developed to detect the discontinuous domains through a comprehensive single-domain library collecting not only from the structure domain databases (SCOP[16] and CATH[9,10]) but also from the sequence domain database, Pfam-A[20–22]. We trained and tested this method on various large-scale datasets and further tested its effectiveness in extending protein domain boundary predictors to detect discontinuous domains through combining DomEX with several domain predictors.

## Methods and Materials

### Domain library

DomEx detects discontinuous-domains by comparing candidate domain sequences with the sequence of known protein domains in the domain library. The DomEx domain library is constructed from three protein domain databases: CATH3.5 [40], SCOP1.75 [41] and Pfam-A. CATH and SCOP are 3D structure databases categorized semi-manually using structural alignment tools. Pfam-A is a curated sequence domain database derived from UniProtKB [42] and containing profile hidden Markov models for sequence search. A pairwise sequence identity cutoff (≥90%) was used to filter out redundant entries from the initial DomEx library, resulting in 5,308,138 domains, where 24,368 domains are from CATH and SCOP and 55,283,770 from Pfam-A. Since the majority of the domains (~99%) are from the Pfam sequence database, the coverage is increased significantly over the structure-only library in ThreaDom.

### Procedure to detect discontinuous domain

DomEx makes three assumptions: (a) Homologous protein domains can be detected by sequence-based profile-profile alignments; (b) Homologous domain pairs have approximately similar length; (c) The coverage and sequence similarity between the different segment pairs in the same homologous domain pairs are usually symmetric. Assumptions (a) and (b) are straightforward. For assumption (c), let some discontinuous domain A has two segments (A_1_ and A_2_) from N- to C-terminal, and it has a homologous domain partner B. The position of the last residue of segment A_1_ is marked as n, then the alignment pair (A-B) could be divided into two segment pairs (A_1_-B_1_ and A_2_-B_2_) at the position between n and n+1. The coverage and sequence similarity of the segment pair A_1_-B_1_ should be close to that of the segment pair A_2_-B_2_. In other words, the coverage and sequence similarity between the two segment pairs are symmetric at the separated point.

Template Similarity Score, Symmetry Index score and Profile-Profile Alignment Score are designed to detect the discontinuous domain. DomEx uses a five-step procedure to assemble and detect the discontinuous domains:

Step 1: Predict the domain/segment boundary positions of a query protein sequence using ThreaDom [38] (or any other domain prediction software).
Step 2: Take all possible nonconsecutive segment pairs as putative discontinuous domains by concatenation.
Step 3: Search the DomEx domain library for hits to homologues templates of the putative domain sequence through a two stage profile alignment with PSI-BLAST.
Step 4: Evaluate the domain assemble score by TS-score, SI and length similarity.
Step 5: Filter the templates from step 4 that are found in Pfam using the profile-profile alignment (PPA) score.
Step 6: Detect conflicts and report the final result.

**Fig 1:**
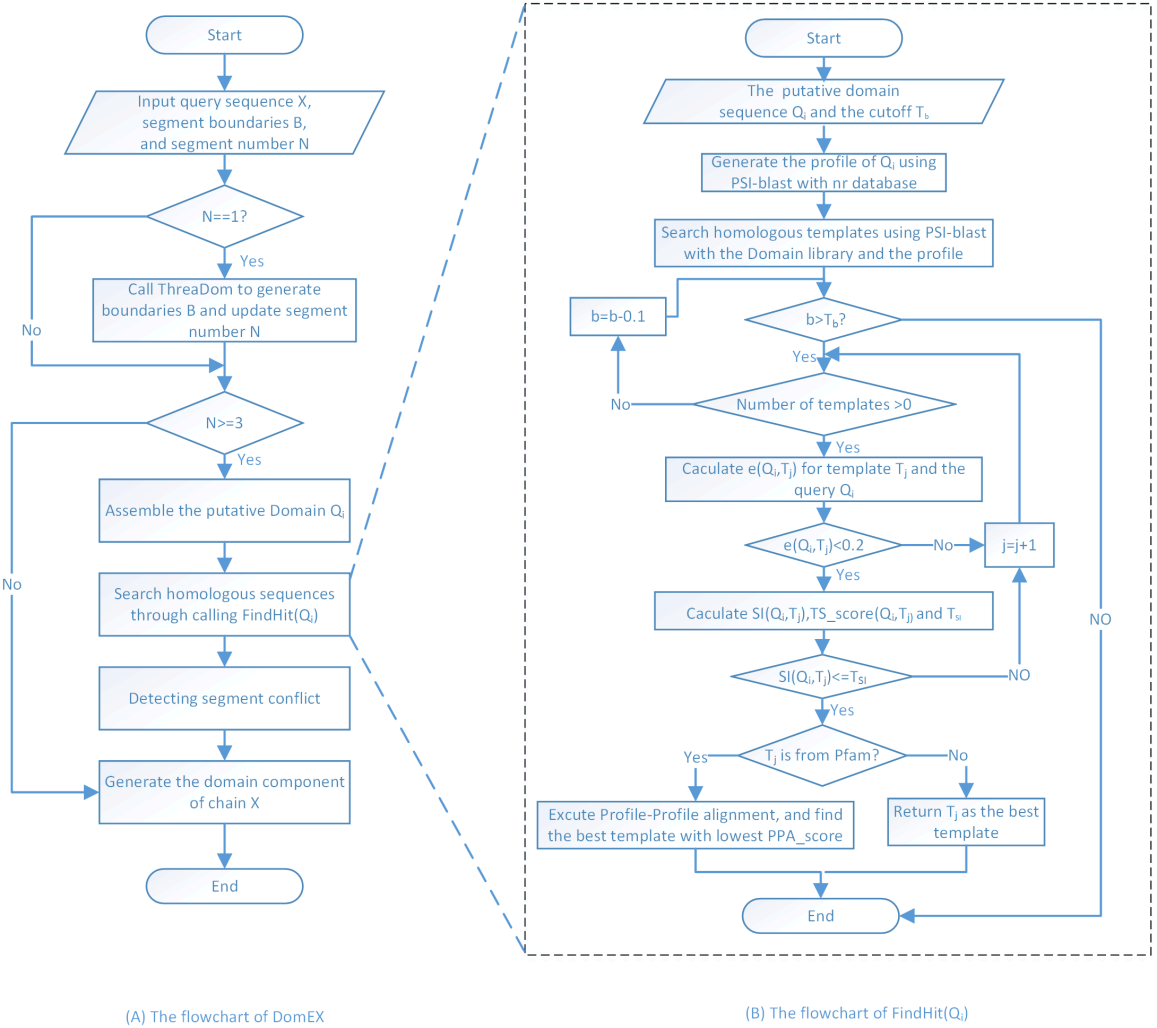
The flowchart of DomEx.

**Fig 2:**
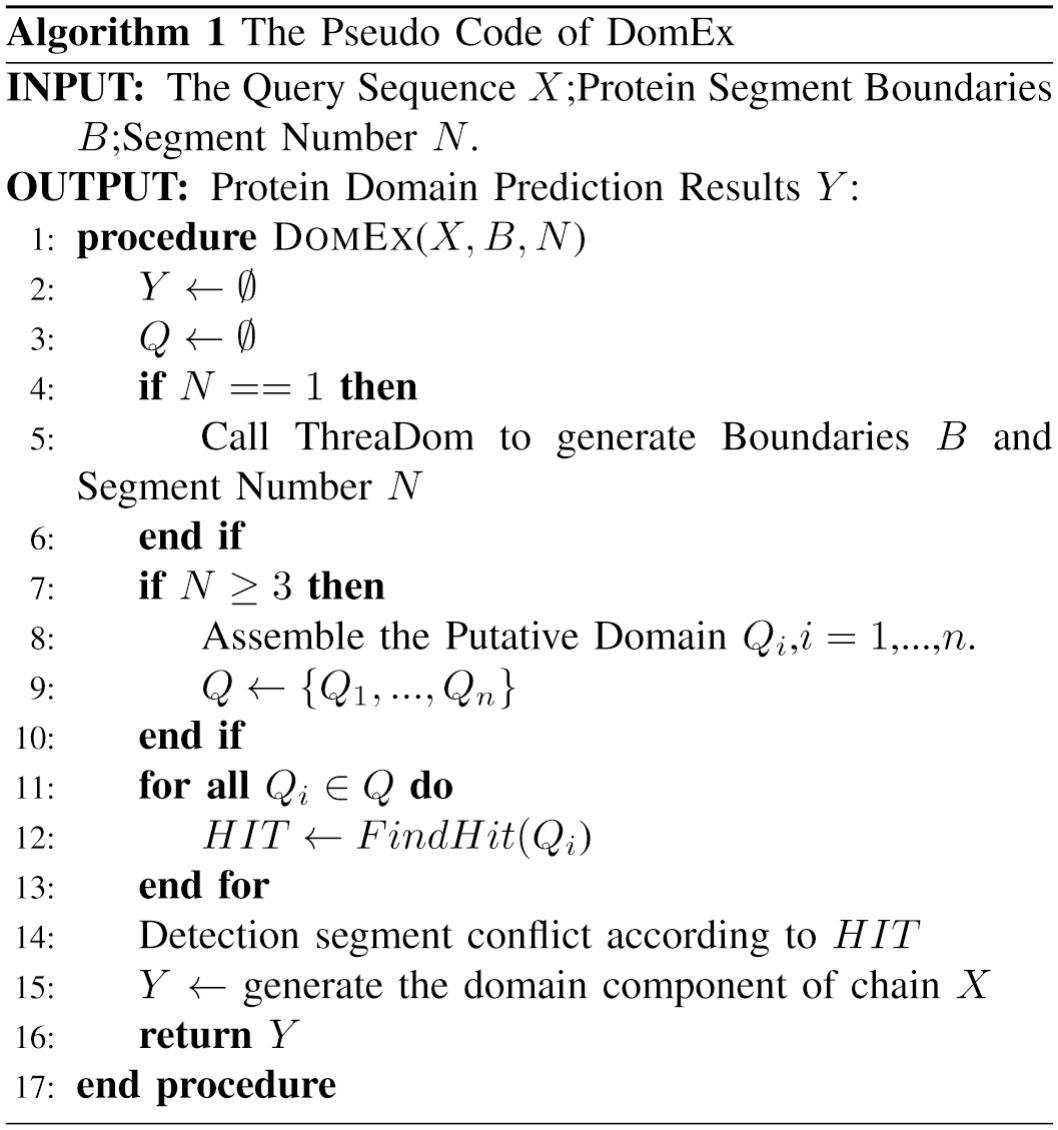
The Pseudo code of DomEx.

**Fig 3:**
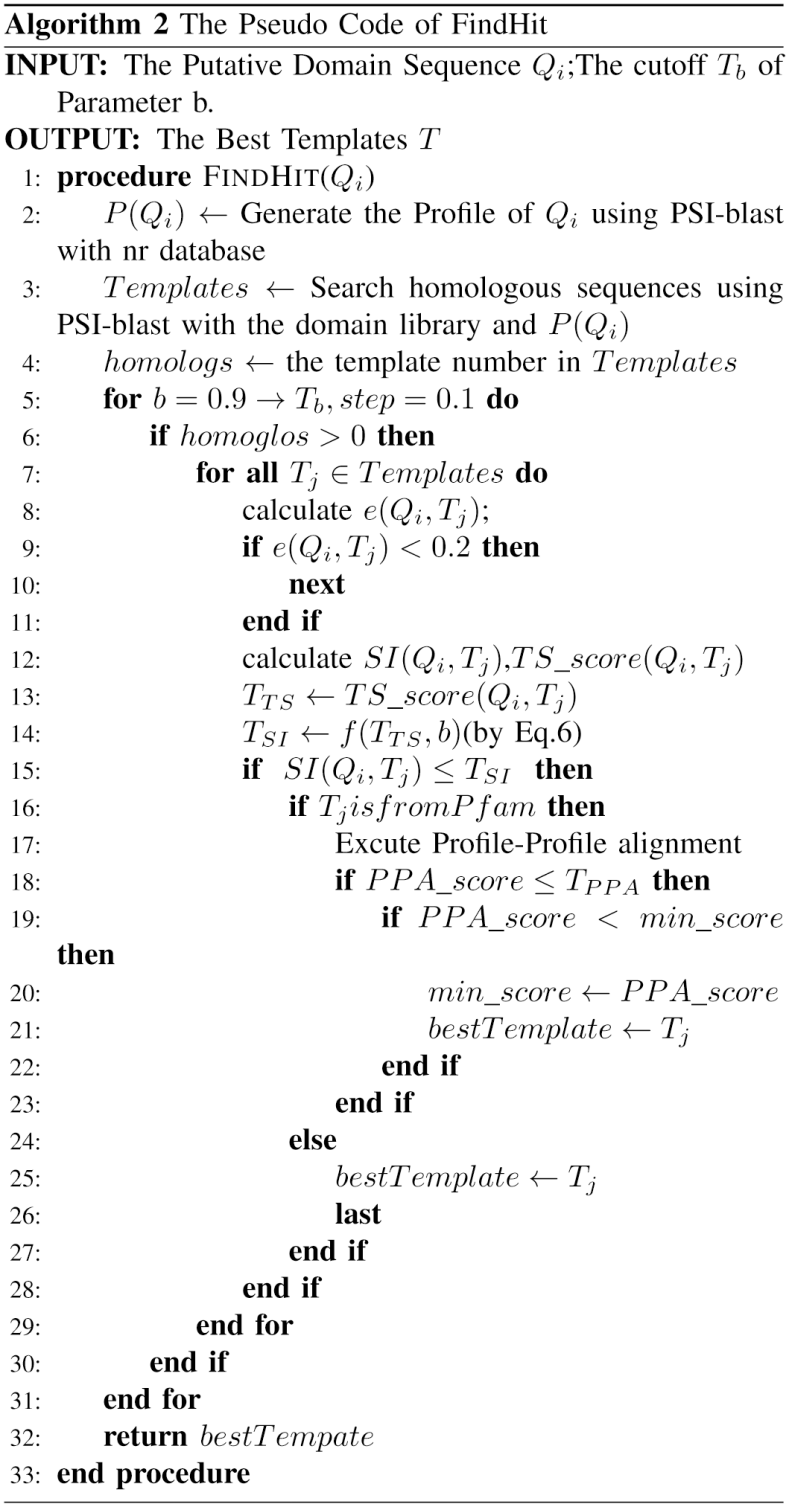
The Pseudo code of FindHit.

The entire flowchart of DomEx is shown in Fig 1. The pseudo code of the main procedure of DomEx is shown in Fig 2. The input consists of the query sequence X, the predicted boundaries B, and the segment number N. DomEx outputs the final detection result by calling FindHit as shown in Fig 3.

An assembled domain sequence *Q*_*i*_ is predicted as discontinuous, if there is at least one hit *T*_*j*_ with length error *e*(*Q*_*i*_, *T*_*j*_)< 0.2, Ts-score(*Q*_*i*_, *T*_*j*_)>*T*_*TS*_ and *SI*(*Q*_*i*_, *T*_*j*_)<*T*_*SI*_. The parameters *T*_*TS*_ and *T*_*SI*_ are the cutoffs of TS-score and Symmetric Index, and they satisfy the constraint function*T*_*SI*_ = *f*(*T*_*TS*_,*b*). This function can be decided by maximizing the Matthews Correlation Coefficient (MCC) value in the training datasets (see below).

If there are multiple candidates that pass through the decision tree, then the candidate with the lowest PPA-score is selected.

### Template similarity score and symmetry index

Consider a protein chain *X* which is divided into *n*+1 segments (S_1_, …, S_n+1_) by n boundary bars (B_1_, …, B_n_), which can be predicted by ThreaDom [38] or any other domain prediction tool. We select two nonadjacent segments *S*_*p*_ and *S*_*q*_ (*p* ≠ *q*), and assemble them into a new putative domain *Q* = (*S*_*p*_∪*S*_*q*_). To examine the possibility that *Q* is a discontinuous domain, we first search the DomEx domain library for hits to some homologous template T of the putative domain sequence through a two stages profile alignment with PSI-BLAST[43], and then we use the Template Similarity score (TS-score) and Symmetric Index (SI) to screen the PSI-BLAST hits.

The TS-score between the query and the template is defined as:

TS-score=s×h×l (1)

where *s* is the sequence identity between Q and the template domain *T* after the alignment. *h* is the normalized E-value from the alignment, i.e. *h* = *min*(*E*_*0*_,−*log*_10_ *E*)/*E*_0_, where *E*_0_ = 10 is the normalization parameter. For example, *h* = 0.3 if the E-value *E* = 0.001, and *h* = 1.0 if *E*≤1*E* − 10. *l* is a factor associated with the alignment coverage (*c*):

$$l=\{\begin{array}{l} 0\text{ if }c\leq 1/3\\ \frac{1}{1+{\left[1/(3c-1)\right]}^{5}\;\}}\;\begin{array}{l} \text{if }c>1/3 \end{array} \end{array}$$ (2)

where *c* equals to the number of aligned residues divided by the length of Q.

To account for the symmetry of the component segments, we define a character vector $\vec{v_{k}}={\left[s_{k}\;c_{k}\right]}^{T}$ for the *k*th segment, where *s*_*k*_ and *c*_*k*_ are the sequence identity and the alignment coverage between the segment of the putative domain and the segment of the template, respectively. A Symmetric Index (SI) between the two segment-pairs is defined as the Euclidean distance between the vectors $\vec{v_{p}}$ and $\vec{v_{q}}$.

$$\text{SI}=∥\vec{v_{p}}-\vec{v_{q}}∥=\sqrt{{\left(s_{p}-s_{q}\right)}^{2}+{\left(c_{p}-c_{q}\right)}^{2}}$$ (3)

To measure the sequence length similarity between the query and template domains, we defined the length variation *e* between the putative query domain (Q) and the template domain (T) from the domain library:

$$e=\frac{\left|L_{T}-L_{Q}\right|}{L_{Q}}$$ (4)

The three parameters of TS-score, SI and *e* will be used to help DomEx find homologous templates from the single-domain library.

### Profile-profile alignment score

The profile-profile alignment combined with the predicted secondary structure information is used to filter out spurious discontinuous domains whose homologous templates are from the Pfam library. The sequence profile and secondary structure prediction are constructed by PSI-BLAST and the consensus of PSSpred (http://zhanglab.ccmb.med.umich.edu/PSSpred) and PSIPRED[44], respectively. The score function is similar to the threading algorithm PPA-I in LOMETS[39]. The profile-profile alignment score is defined as the score of the best alignment from dynamic programming between the query and template.

### Training, validation and testing datasets

We constructed three datasets including Training Dataset, Validation Dataset and Testing Dataset. Training Dataset and Validation Dataset are used to train and validate the parameters of DomEx. In the training procedure, holdout validation is employed. The Validation Dataset is independent of the Training Dataset. The Testing Dataset is used to compare DomEx with ThreaDom. Furthermore, the Testing Dataset is also used to test the performance of detecting the discontinuous domain when DomEx is combined with other domain predictors.

**Fig 4:**
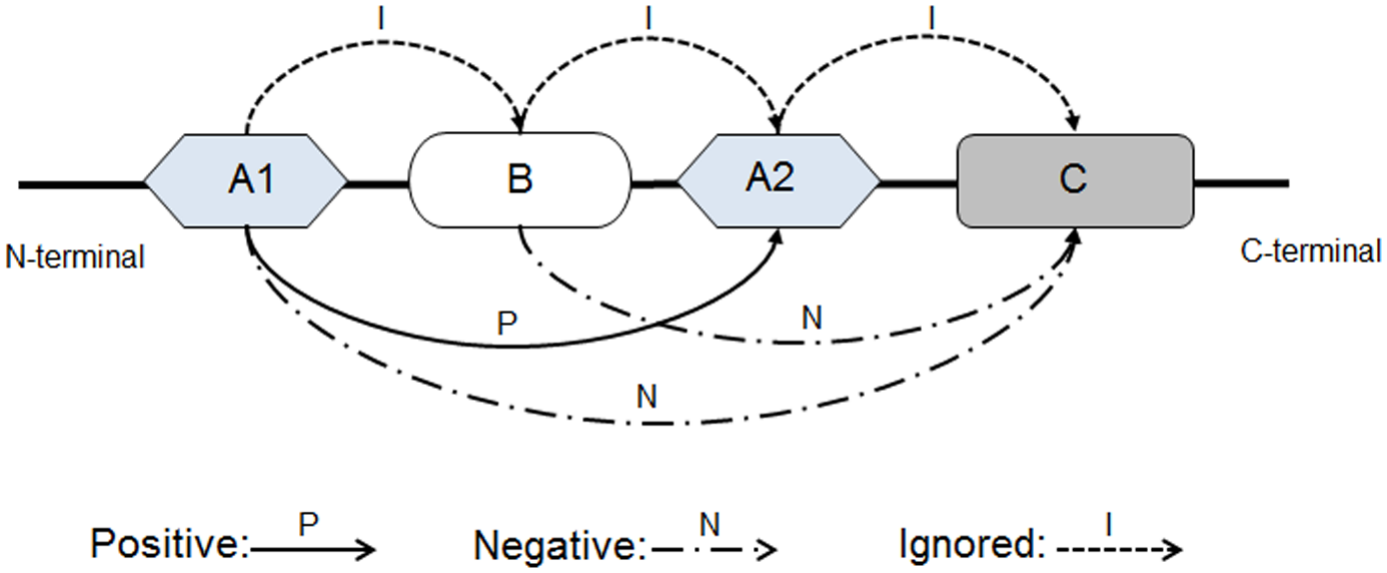
An illustration of the procedure to generate the samples. A 3-domain chain is defined as (A1A2)(B)(C). A1 and A2 form one structure domain, while B and C are independent domain, respectively. The (A1A1) is treated as “Positive” sample; (A1C) and (BC) as “Negative” and other combinations are ignored.

The “Positive” and the “Negative” domain samples in the Training and the Validation Datasets are derived from the known structure domain segments. A positive sample refers to the segment combination that constitutes a true structure domain, while a negative sample is the combination that does not constitute a structural domain. Fig 4 shows an example of protein chain consisting of four segments that form three domains: (A1A2)(B)(C). Segment A1 and A2 form one structure domain; B and C form the other two independent domains. Then the segment assembly (A1A2) is a “Positive” sample, while (A1C) and (BC) are treated as “Negative” samples. Combinations of adjacent segments combination such as (A1B), (BA2), (A2C) are ignored as they are neighboring in sequence. The reversed combinations from the C- to N-terminal, e.g. (BA1) and (CB), are also ignored here, but will be discussed in the discussion section. Only the discontinuous domains containing two segments were considered here, since discontinuous domains including more than three segments are very rare (<2% in CATH3.5), and the extension to three-segment domains is straightforward.

From the CATH3.5 library, we collected 481 non-homologous proteins, which have known domain structure and consist of at least three segments. Among them, 326 contain at least one discontinuous domain and 155 have three or more continuous domains. The pairwise sequence identity between the proteins is below 25%. From these proteins, we generated 344 positive discontinuous domains and 822 negative samples. The 822 negative samples have 273 from continuous multi-domain chains and 549 from incorrect discontinuous domain segment assemblies. Here, we only consider the cases that the segments have at least 40 residues, because most of protein domain predictors[28,31,32,38] consider a prediction to be “correct” if the predicted boundaries are within ±20 residues away from the true boundary. Then the maximum error of a correctly predicted segment is 40 residues and these domain predictors cannot report the domain boundary when the protein domain is less than 40 residues according to this criterion.

From the segment assemblies, we randomly selected 229 positive and 548 negative samples which are used as the training dataset to decide the parameters *T*_*TS*_ and *T*_*SI*_; the others are used as the validation dataset to test the parameters obtained from training.

Our test set includes two subsets TEST-SET-I and TEST-SET-II. TEST-SET-I is used to test the robustness of DomEx by comparing it to ThreaDom alone on discontinuous domain detection. It contains 97 discontinuous domain protein chains, and all the boundaries predicted by ThreaDom are within ±20 residues to the annotated boundaries, and 80% of the boundary predictions have the error within ±5 residues.

TEST-SET-II is used to benchmark the domain predictors. It contains the same chains from which the training and the validation datasets were derived, but the boundaries will be predicted by different predictors.

### Evaluation

The standard measurements of recall, precision and Matthews Correlation Coefficient (MCC) are employed to evaluate the performance of detecting the assembly of discontinuous domains from segments:

$$\{\begin{array}{l} recall=\frac{TP}{TP+FN}\\ precision=\frac{TP}{TP+FP}\\ \begin{array}{l} \text{MCC}=\\ \frac{TP\times TN-FP\times FN}{\sqrt{\left(TP+FP)(TP+FN)(FP+TN)(TN+FN\right)}} \end{array} \end{array}$$ (5)

where TP, FP, TN and FN denote the number of true positives, false positives, true negatives and false negatives, respectively.

NDO-score [45]is used to benchmark the different protein domain predictors. The NDO-score is defined as the normalized overlap rate of all predicted domain and linker regions with the true domain assignment in the native structure.

## Results

### Training and validation of DomEx

We trained DomEx using a 3-stage strategy: Exhaustive Search Training (EST), Equation Constraint Validation (ECV) and PPA Check Training (PCT). The EST procedure is used to train the cutoff *T*_*TS*_ of the TS-score and the cutoff *T*_*SI*_ of the SI with the Training Dataset. The ECV procedure is used for tuning the correlated cutoff parameter *b* in the constraint function *T*_*SI*_ = *f*(*T*_*TS*_,*b*) based on the Training Dataset. PCT is used to train the PPA-score cutoff *T*_*PPA*_ which is used to filter out incorrect templates from Pfam using the profile-profile alignment. All the parameters are validated on the Validation Dataset based on the holdout validation.

#### Exhaustive search training (EST)

**Fig 5:**
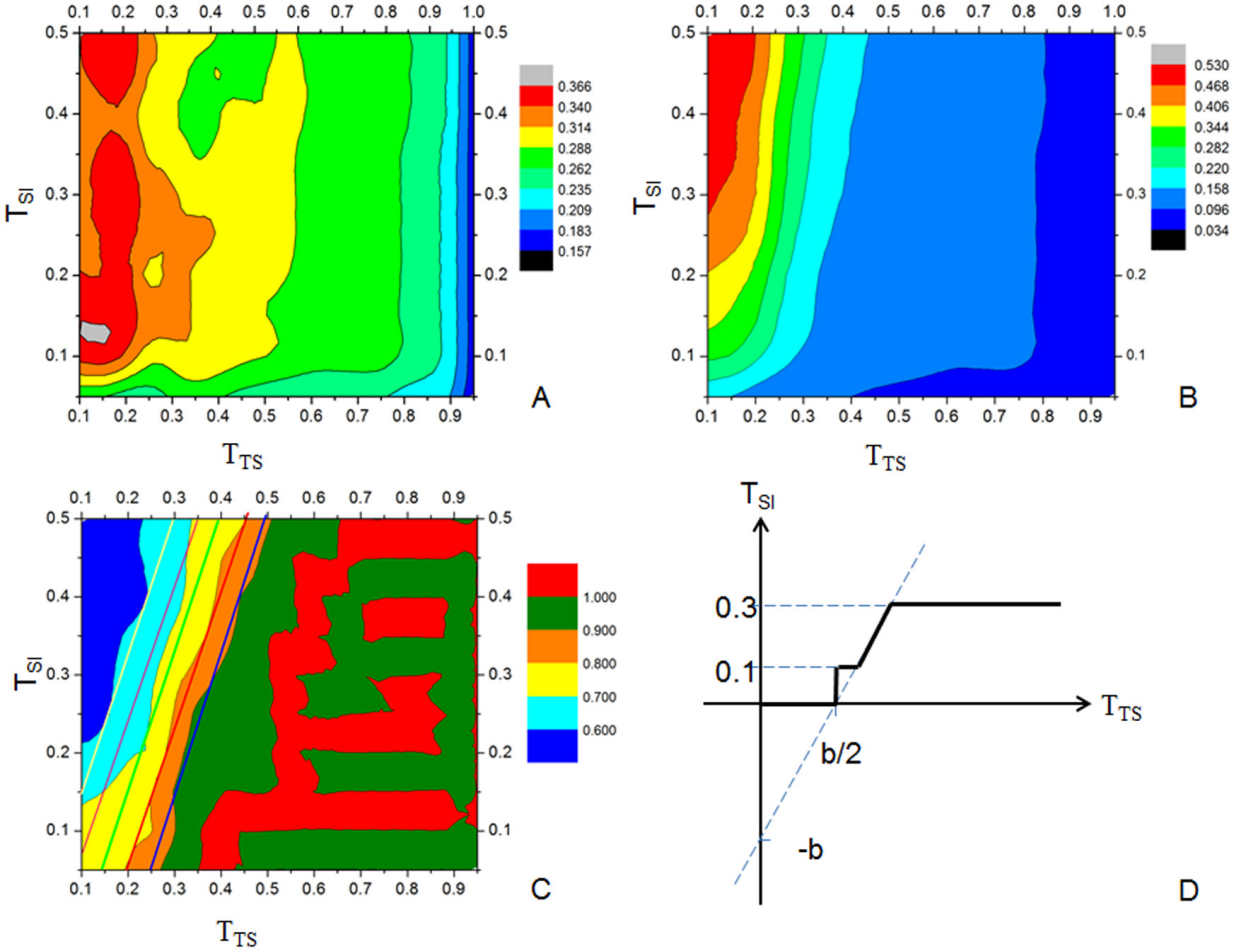
The recognition results of discontinuous domains at various TS-score and SI cutoffs. (A) MCC; (B) Recall; (C) Precision (5 parallel lines show the boundaries of the precision region at different b values). (D) The figure of Eq 6.

An exhaustive grid search of the 2-dimensional space of TS-score and SI was performed to find the best combination of cutoffs *T*_*TS*_ (for TS-score) and *T*_*SI*_ (for SI). Using a step size of 0.05, we searched for the optimal values of *T*_*TS*_ and *T*_*SI*_ in the range [0.1, 1] and [0.05, 0.5], respectively. The average MCC, recall and precision for the training dataset with different *T*_*TS*_ and *T*_*SI*_ are shown in Fig 5A–5C, respectively. It is easy to see that a high TS-score cutoff usually has high precision but low recall. As shown in Fig 5A to 5C, the boundaries of the adjacent regions are close to vertical lines when TS-score >0.5, which indicates that the MCC, recall and precision values are not sensitive to the Symmetric Index in the region of high TS-score.

The Symmetric Index becomes more important when TS-score <0.5. The highest MCC values (> 0.3) are in the region of TS-score ∈[0.1, 0.2] and SI∈[0.1, 0.2], where DomEx has a reasonable precision (>0.6) and maximum recall (>0.34).

#### Equation constraint validation (ECV)

Since there is no unique cutoff that can achieve the best MCC, we designed a constraint relationship function *T*_*SI*_ = *f*(*T*_*TS*_,*b*) with a dynamic relationship between the cutoff *T*_*TS*_ and the cutoff *T*_*SI*_ controlled by a single parameter b, i.e.

$$T_{\text{SI}}{\text{=f(T}}_{\text{TS}}\text{,b)=}\{\begin{array}{l} \text{0}.\text{3},\;0.15+\text{b}/2<T_{TS}\\ {\text{2T}}_{TS}\text{-b},\;0.05+\text{b}/2<T_{TS}<0.15+\text{b}/2\\ 0.1,\text{ b}/2<T_{TS}<0.05+\text{b}/2\\ \text{0},{\text{ T}}_{TS}<\text{b}/2 \end{array}$$ (6)

where different values of b correspond to different parallel lines that separate regions with similar precision values in Fig 5C. The curve of Eq 6 is shown in Fig 5D, which ensures that SI stays in the favorable region of [0.1, 0.3]. For example, given a parameter b = 0.4, a query sequence is aligned to some template with TS-score = 0.30 and SI = 0.15, then let TS-score≥*T*_*TS*_ = 0.30, and we get *T*_*SI*_ = 0.2 according to the Eq 6. We can infer that this query is a discontinuous domain because TS-score≥*T*_*SI*_ = 0.30 and SI≤*T*_*SI*_ = 0.2. Algorithm 2 (Fig 3) also shows this calculation procedure.

**Fig 6:**
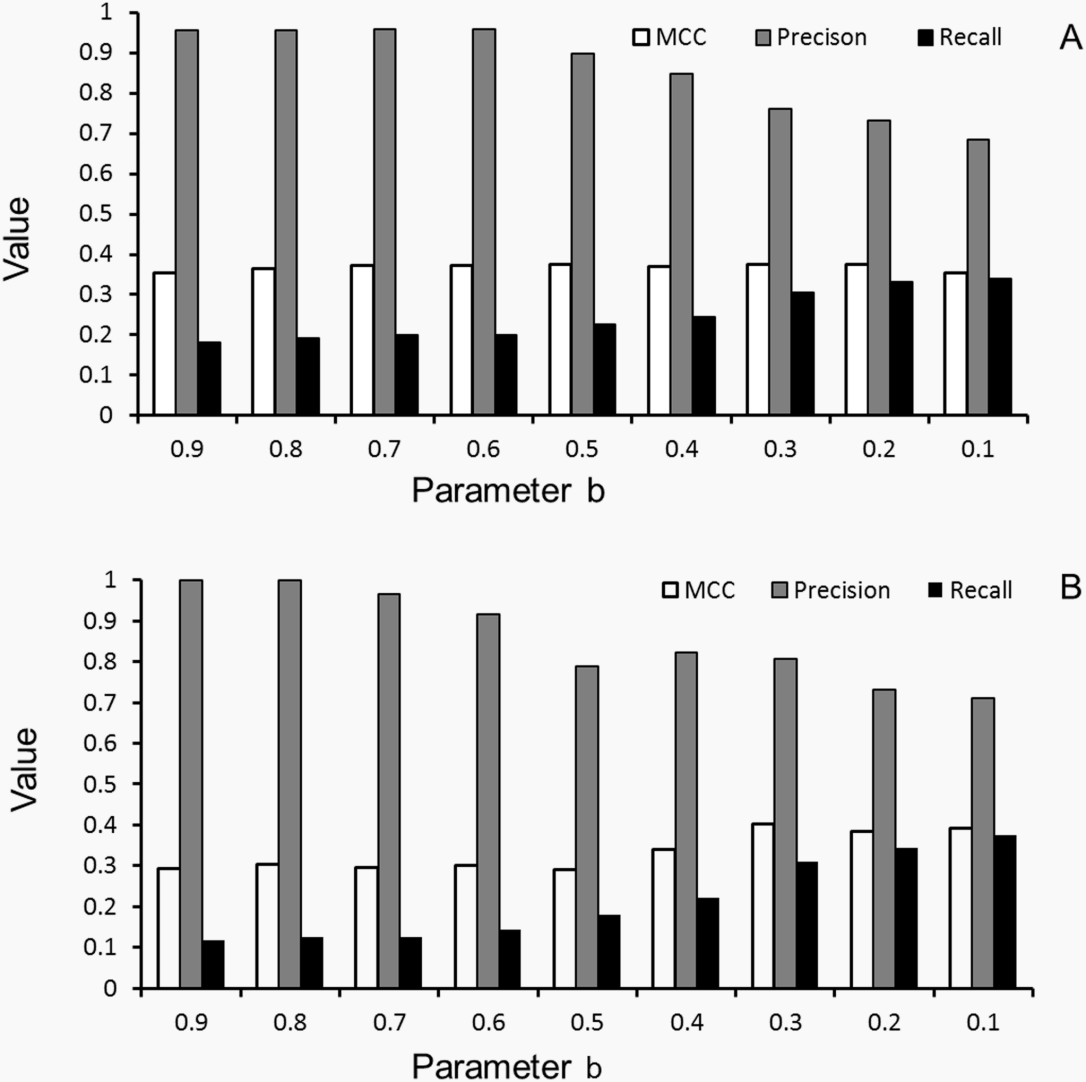
The training and validation results using *T*_*SI*_ = *f*(*T*_*TS*_,*b*) constraints. The cutoff *T*_*TS*_ (for TS-score) and *T*_*SI*_ (for SI) in Fig 5 are constrained by Eq 6. (A) The results on the Training Dataset with parameter b from 0.9 to 0.1; (B) The results on the Validation Dataset with parameter b from 0.9 to 0.1, respectively.

As confirmation, DomEx can achieve a reasonable MCC prediction above 0.3 for all the b values not only on the Training Dataset (Fig 6A), but also on the Validation Dataset (Fig 6B). It is more robust than that in EST step.

#### PPA check training (PCT)

The training results were rechecked by Profile-Profile Alignment (PPA). The PPA-score gives the alignment quality between the query and template sequence. The score is usually negative. A low score means a good alignment. In the ECV training, there are 109 out of the 288 positive samples correctly detected as discontinuous, and 72 out of the 548 negative samples incorrectly detected as discontinuous when b>0.1. We use a total of 181 alignments as input to train the best PPA-score cutoff. We found the PPA can improve the MCC and precision when b<0.5 and PPA-score <-1.90 when the templates are collected from Pfam. Then the query is treated as a “Positive” detection, when the parameter b<0.5 and the PPA-score <-1.90.

We tested the method on 390 discontinuous-domain samples on the Validation Dataset, using b from 0.9 to 0.1 with step -0.1 and PPA-score <-1.90 when b<0.5. Similar to the tendency on the Training Dataset, the precision of DomEx in the Validation Dataset varies from 1.0 to 0.771. The MCC values are all higher than 0.30 with recall ranging from 0.183 to 0.339. For better balance between precision and recall, we selected b = 0.3 and PPA-score cutoff *T*_*PPA*_<−1.90 as the default parameters in DomEx.

### Test of DomEx

#### Discontinuous domain detection using accurately predicted boundaries

To detect the discontinuous domains, DomEx depends on the predicted boundaries from other predictors, such as ThreaDom. The test dataset TEST-SET-I contains 99 positive and 58 negative discontinuous domains which were derived from a non-redundant set of protein chains consisting of 97 discontinuous domains (Identity<25%, the length of shortest segments >40). Each boundary was predicted by ThreaDom within an error of ±20 residues to the boundary definitions in CATH 3.5.

In Table 1, we summarize the prediction results according to MCC, recall and precision and compare the results with the discontinuous domain detection of ThreaDom. DomEx recalled 32% of the discontinuous domains with 86% precision, which is similar to the results of using the annotated segment divisions from CATH 3.5. The discontinuous-domain detection method of ThreaDom is based on clustering the boundaries of the discontinuous domain templates. It is not surprising that it achieves a recall of 67.7% and precision of 87%, which is much higher than DomEx because ThreaDom is structured-based. But DomEx uses both the structure and sequence-based libraries, so it can handle the cases without 3D templates, where ThreaDom failed. We found that there are 29 chains where ThreaDom failed to detect the discontinuous domains (Group II in Table 1). However, for these chains, DomEx recalled 26.7% of the discontinuous domains with 72.7% precision. Half of the correct sequence templates came from Pfam, which demonstrates that DomEx works when there are no templates with known 3D structure.

**Table 1:** Discontinuous domain detections from predicted boundaries by ThreaDom.

Group	Prediction results			
	Method	Recall	Precision	MCC
**^a^** **Group I**	DomEx	0.348	0.923	0.271
**Group I**	ThreaDom	1.000	1.000	1.000
**^b^** **Group II**	DomEx	**0.267**	**0.727**	0.226
**Group II**	ThreaDom	---	---	---
**ALL**	DomEx	0.323	0.865	0.270
**ALL**	ThreaDom	0.677	0.870	0.568

DomEx predicted discontinuous domains with the boundaries predicted by ThreaDom boundary prediction method. ThreaDom predicted discontinuous domains with its own discontinuous domain detection method and ThreaDom boundary prediction method.

^a^Group I: The subset of TEST-SET I with 69 positive samples and 26 Negative samples from 68 protein domain chains that ThreaDom detects the discontinuous domain correctly.

^b^Group II: The subset of TEST-SET I with 30 positive samples and 32 Negative samples from 29 protein domain chains that ThreaDom fails to detect the discontinuous domain.

#### Discontinuous domain prediction using TEST-SET-II

As a control, we employed five publicly available domain predictors, including ThreaDom[38], FIEFDom[26], DomPro [28], DROP[31] and PPRODO[30], which represent different types of homology- and machine-learning-based methods. Among them, ThreaDom can detect discontinuous domains, while the others cannot.

To test the situation where there are only weakly homologous templates, for ThreaDom, we excluded all templates that have a sequence identity >30% to the target protein, and we also excluded templates that are detectable by PSI-BLAST with an E-value<0.05.

For FIEFDom, we kept two group boundary prediction results, FIEFDom I and FIEFDom II. FIEFDom I excludes the templates if their sequence identity >30%; while FIEFDom II includes all the templates.

**Fig 7:**
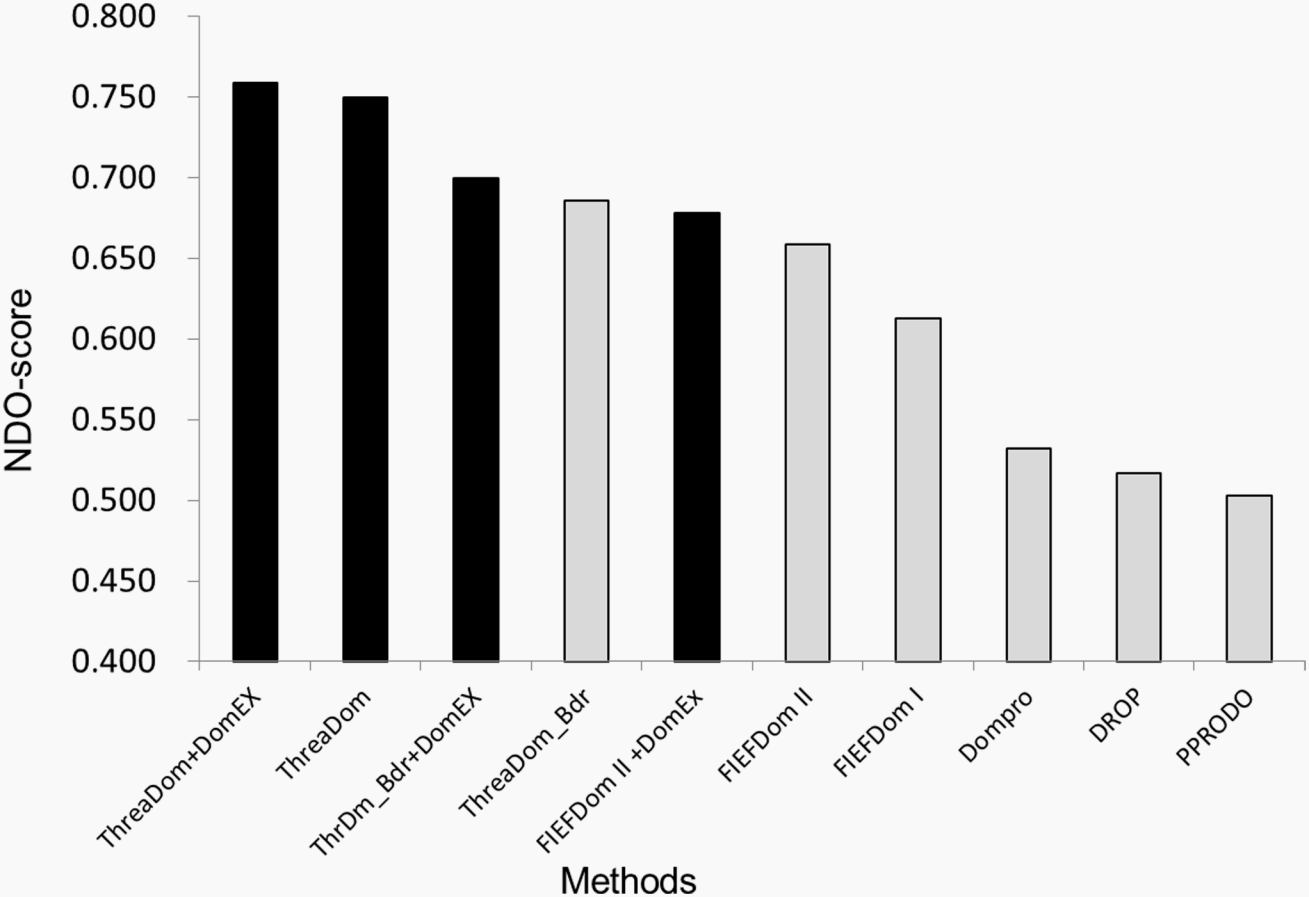
The benchmark results of DomEx with domain boundary predictors. The methods with discontinuous domain detection are shown as dark bars.

The dataset TEST-SET-II contains 481 multi-domain chains, which is the same size as the training and validation sets. It includes 326 discontinuous chains and 155 continuous domain chains. Each chain has at least 3 segments, and the length of each segment is not less than 40 residues. Fig 7 illustrates the NDO-score of the different methods. Here, we chose the best two boundary predictors, ThreaDom_Bdr and FIEFDom II to benchmark the performance of DomEx. They are denoted as ThrDm_Bdr+DomEx and FIEFDom II+ DomEx, respectively. We used ThreaDom_Bdr to represent the method which only predicts the boundaries without the boundary optimization and discontinuous domain detection option of ThreaDom. ThreaDom+DomEx uses DomEx to detect discontinuous domains when ThreaDom does not detect any discontinuous domains. In Fig 7, the dark bars represent the methods that support discontinuous-domain detection.

When detecting the discontinuous domains, both DomEx with ThreaDom boundaries (ThrDm_Bdr+ DomEx) and DomEx with FIEFDom II boundaries (FIEFDom II+DomEx) have a higher NDO-score than their boundary prediction without DomEx detection. And the NDO-score of ThrDm_Bdr+DomEx (0.70) is higher than all the predictors that do not support discontinuous-domain detection. ThreaDom+DomEx has the highest NDO-score of 0.759. The results demonstrate that DomEx can improve the discontinuous domain detection when combined with other boundary predictors because of the addition of a sequence-based domain library and the symmetric alignment score.

#### Test DomEx with CASP targets

There are a total of 17 targets which have > = 3 continuous segments (length > 30 residues) from CASP8 to CASP10 experiments. Six contain multiple continuous domains, and eleven contain at least a discontinuous domain. A summary of the domain definitions from the CASP assessors is listed in S1 Table. To eliminate the negative effect of inaccurate boundary prediction, the assessor-based boundaries were used as input. The result showed that 36.7% discontinuous-domain proteins were correctly detected.

## Discussion

### Effect of templates from SCOP+CATH or Pfam

**Fig 8:**
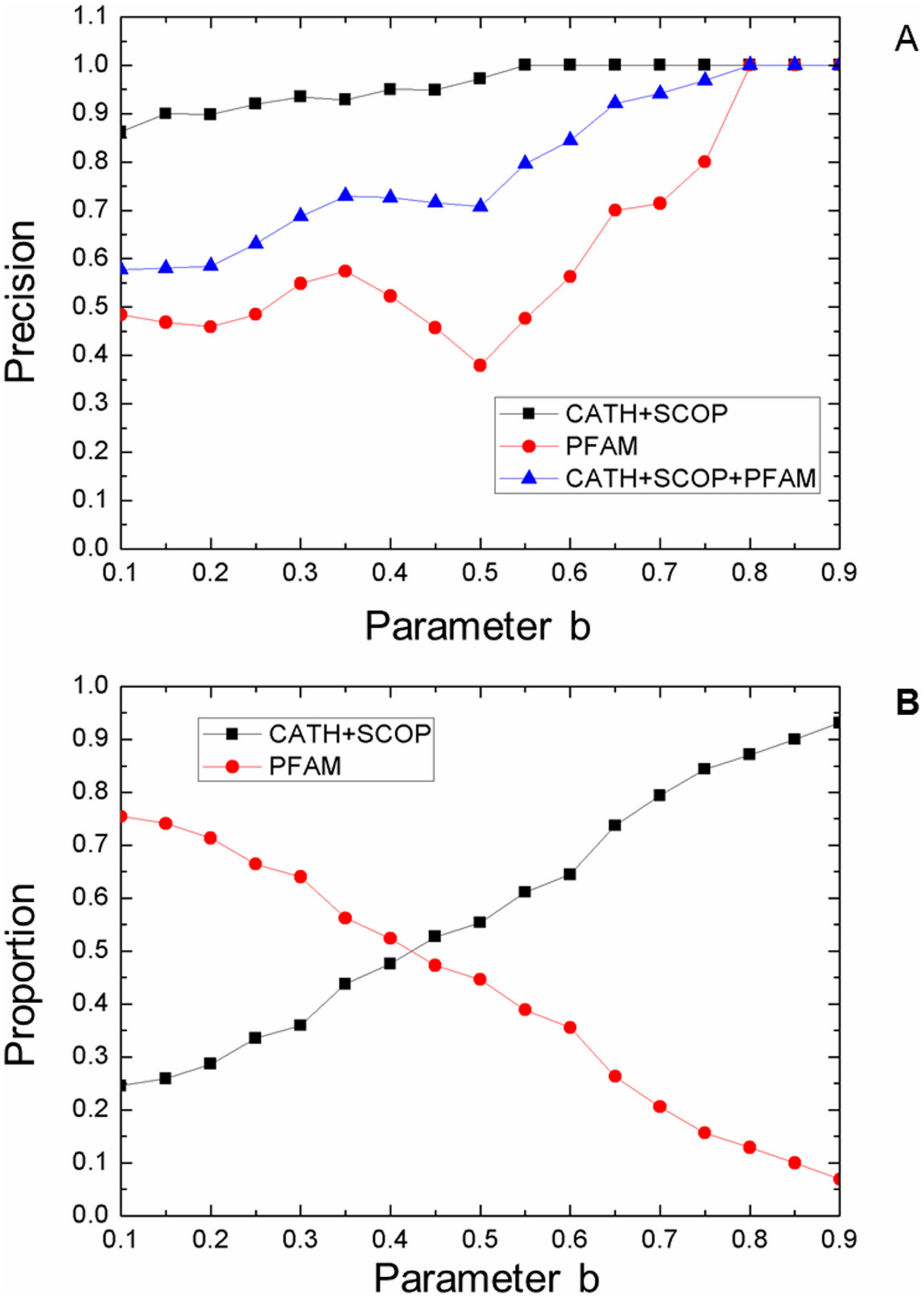
The comparison of the templates from CATH+SCOP, Pfam and CATH+SCOP+PFAM. (A) Precision comparison; (B) The proportion of templates coming from CATH+SCOP and Pfam as parameter b varies.

The domain sequence library of DomEx is based on CATH, SCOP and Pfam-A. The domain boundaries of SCOP and CATH are defined based on the 3D structure of the proteins. Pfam-A is based on the HMM classification of sequences from whole-genomes. It is observed that the accuracy of templates from the Pfam library is about 60~70% of that from CATH+SCOP. Fig 8A shows the comparison of the templates from CATH+SCOP, Pfam and CATH+SCOP+Pfam. Given a high b (for example, *b*>0.8), DomEx achieves a high precision and is independent of the template sources (CATH+SCOP or Pfam). When *b*<0.8, the accuracy of the Pfam-based prediction is significantly lower than CATH+SCOP. If the template comes from CATH or SCOP, DomEx has a high precision (>90%). If the templates come from Pfam, the precision declines to about 50% when b is less than 0.65.

Fig 8B gives the proportion of the templates from CATH+SCOP and Pfam across different values of *b*. For large *b*, the templates come mainly from CATH and SCOP, while for small *b*, much more templates are from Pfam. (We also note that the datasets are collected from CATH3.5, but the results are more convincing because the domain boundary definitions are based on known 3D structures) When *b* = 0.2, about 70% of the templates come from Pfam, and the recall of DomEx is about 44%. The recall is 100% higher than when *b* = 0.5, where 50% of the templates come from Pfam. The templates from Pfam increase the recall of DomEx, even though the precision of DomEx decreases when b decreases. The domain sequences from Pfam improve recall because of its large size relative to the other databases.

### Effect of PPA score

In DomEx, the Profile-Profile Alignment combined with the predicted secondary structure information is used to help DomEx improve the low precision when the templates are from Pfam. Table 2 compares three PPA checking strategies: 1) with PPA check of only the templates from Pfam (Group I), 2) with PPA check of the templates from CATH+SCOP+Pfam (Group II), and 3) without PPA check (Group III). The PPA-score cutoff *T*_*PPA*_ in Table 2 is the one which has maximum MCC for some *b* when *T*_*PPA*_ varies from 0.0 to -5.0 with step -0.1. The average MCC and precision of Group I, which are 0.341 and 0.901, respectively, are higher than the other two. From the table, one can see that Group I has higher recall and MCC values for the cases when the precision values are similar to the other groups (underlined in table). For example, Group I has precision of 80.6% at *b* = 0.3, which is a bit higher than Group II at *b* = 0.4 and Group I at *b* = 0.5, while its recall is 31% and 61% higher than the other two, respectively.

**Table 2:** Comparison of the Different PPA Checking Strategy.

b	With PPA check only to Pfam templates (Group I)				With PPA check to all type templates (Group II)				Without PPA check (Group III)		
	T_PPA_	Recall	Precision	MCC_best	T_PPA_	Recall	Precision	MCC_best	Recall	Precision	MCC
0.9	0.0	0.118	1.000	0.294	-0.15	0.118	1.000	0.294	0.122	1.000	0.299
0.8	0.0	0.127	1.000	0.305	-0.15	0.127	1.000	0.305	0.131	1.000	0.310
0.7	0.0	0.127	0.967	0.295	-0.15	0.131	0.968	0.301	0.135	0.969	0.306
0.6	-3.20	0.1354	1.0000	0.3153	-2.20	0.140	0.941	0.303	0.153	0.897	0.304
0.5	-3.30	0.1572	0.9730	0.3327	-1.50	0.179	0.820	0.302	0.192	0.786	0.300
0.4	-2.90	0.1921	0.9167	0.3500	-1.50	0.236	0.794	0.339	0.253	0.753	0.334
0.3	-1.90	**0.3100**	**0.8068**	**0.4014**	-1.50	0.332	0.768	0.396	0.358	0.739	0.398
0.2	-1.90	0.3450	0.7315	0.3849	-1.50	0.371	0.675	0.367	0.406	0.620	0.349
0.1	-1.90	0.3755	0.7107	0.3919	-1.50	0.424	0.660	0.387	0.476	0.602	0.372
Average	--	0.210	**0.901**	**0.341**	--	0.229	0.847	0.333	0.247	0.818	0.330

PPA filtering is not as effective for Group II as it is for Group I. The main reason is the domain definitions from SCOP and CATH are more likely to be correct than Pfam since they are derived from known 3D structures. Therefore, we used PPA filtering only for templates from Pfam library.

### Reversed assembly

In the segment assembly, we considered only the segment orderings from N- to C-termini, e.g. (A1A2) in Fig 4. We also examined the possibility of reversed segment assembly, e.g. (A2A1) in Fig 4, where the order of segments is reversed but the residue order within each segment sequence is unchanged. We found that DomEx reported only 3 discontinuous domains out of all the 344 positive samples in the training and validation dataset using the reversed order when b = 0.2, which is less than 0.9% of the positive samples. Therefore, we have ignored the reverse assembly in the default settings of DomEx to save CPU time (about twice as long).

**Fig 9:**
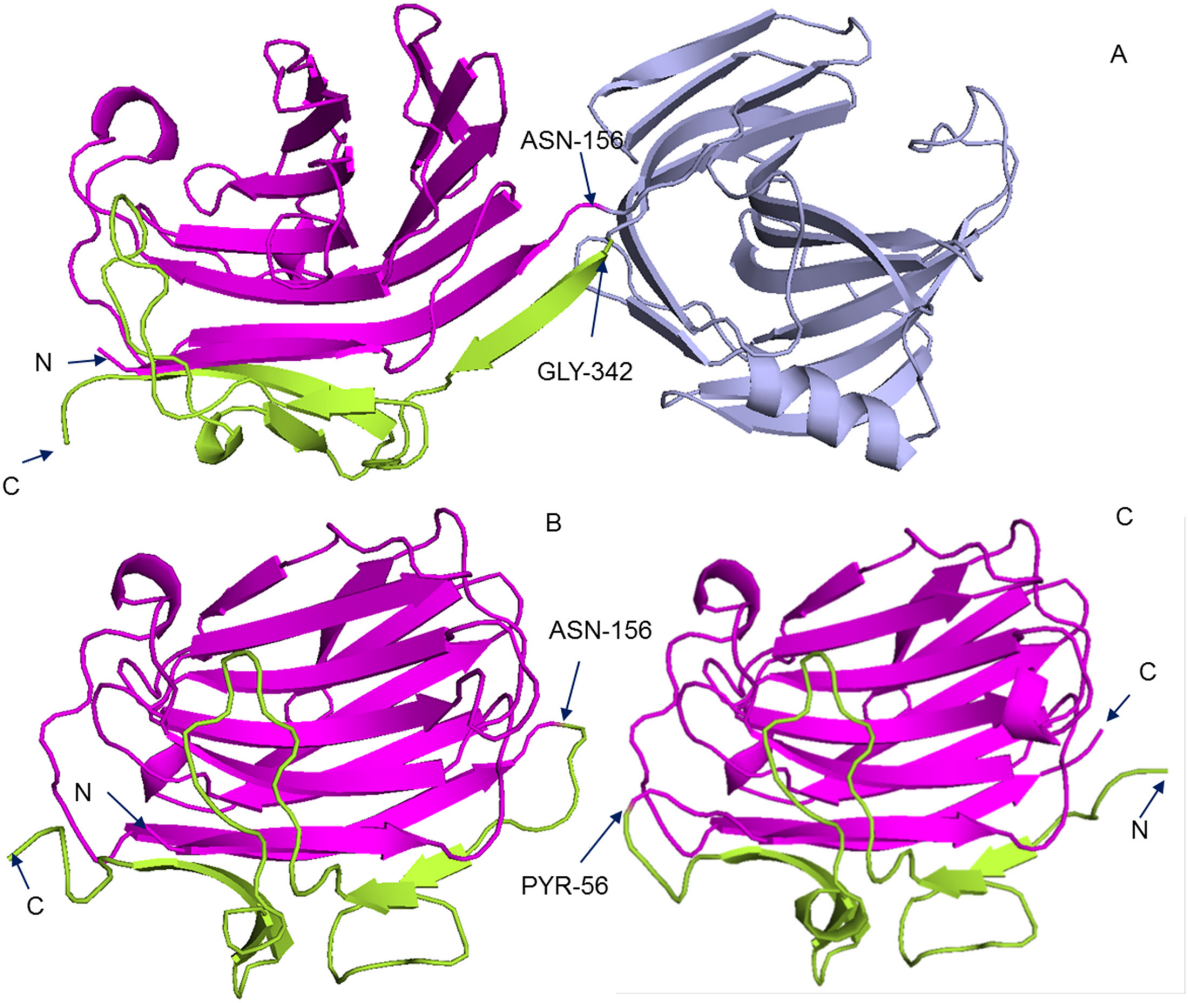
The cases of N- to C-termini assembly. (A) The 3D structure of PDB: 1axkB. The two segments of the discontinuous domain (1–156|342–393) are colored in magenta and lemon green, respectively. (B) The 3D structure of PDB:1u0aD. It is an AB type Segment-Swapping Domain. (C) The 3D structure of PDB:1cpmA. It is a BA type Segment-Swapping Domain.

Nevertheless, the reversed segment assembly can detect some interesting domain structures. All the three reversed cases are from proteins with segment-swapping domains (SSDs) [46]. There are two cases the templates coming from CATH and one case from Pfam. Fig 9 shows two examples with templates from CATH3.5 whose structure is known. In Fig 9A, the template has the PDB ID of 1axkB which is defined as (1–156|342–393)(157–341) in CATH3.5. The first domain is discontinuous, and the two segments are colored in magenta and lemon green, respectively. Fig 9B and 9C show the templates (PDB ID: 1u0aD and 1cpmA) for the same sequence, which are detected by segment assembly using orders from N- to C-termini and from C- to N-termini, respectively. The two templates are both single-domain chains, where 1u0aD is an AB type SSD and 1cpmA is a BA type SSD. They both have similar 3D structure. In the DomEx package, we have included an option to turn on reversed segment assembly.

## Conclusion

We have proposed a new strategy, DomEx, to extend the ability of domain boundary predictors to detect discontinuous domains. The method assembles and detects discontinuous domains from the sequence segments. DomEx incorporates template similarity, symmetry of segment pairs, profile-profile alignments, and structure-based and structure-free libraries.

Two test benchmarks showed that DomEx not only worked with the boundary predictors, but also was complementary to the discontinuous-domain detection method in ThreaDom. DomEx recalled 26.7% of the cases where ThreaDom failed. Half of these cases are attributed to templates from Pfam. When compared to other methods, DomEx plus ThreaDom gave the best NDO score, which further confirms that DomEx can detect discontinuous domains even without known 3D-structure templates. The main advantage of DomEx is that it searches for templates using domain-domain alignments rather than chain-chain alignments. Using domain-domain alignments improves recall because chain-chain alignments may miss templates where the domains match, but the rest of the chain does not match well. The benchmark results show that DomEx is an effective method, which opens the possibility of finding discontinuous domains in genome-wide studies. Currently, DomEx supports the detection of two-segment discontinuous domains. Further work will extend the model to detect discontinuous domains with more than 2 segments, and try to utilize the domain annotated information from CATH, SCOP and Pfam to enhance the performance of detecting discontinuous domains. The accuracy of boundary predictors and sequence alignment tools should also improve the detection results. The source code and datasets of DomEx are available at https://github.com/xuezhidong/DomEx.

## Supporting Information

S1 Table Domain definition of the 17 targets in CASP8, CASP9 and CASP10 to test DomEx. (PDF)
