# Supplementary material for: Extending Protein Domain Boundary Predictors to Detect Discontinuous Domains
Source: PLoS One. 2015 Oct 26;10(10):e0141541. doi: 10.1371/journal.pone.0141541 (PMC4621036; doi:10.1371/journal.pone.0141541)
Supplement: S1 Table — (PDF) [file pone.0141541.s001.pdf]

Supporting Information

S1 Table. Domain definition of the 17 targets in CASP8, CASP9 and CASP10 to test DomEx

Targets	Sequence Length	Domain Number	Domain Definition
T0416	332	2	(6-119|194-315)(124-183)
T0431	491	2	(7-79|344-372)(80-343|373-488)
T0445	264	2	(1-81|191-264)(83-189)
T0472	110	2	(1-51|102-110)(52-101)
T0504	208	3	(1-62)(63-154)(157-208)
T0505	290	2	(6-84|201-280)(86-199)
T0510	288	3	(1-165)(166-235)(236-279)
T0521	179	2	(1-34|107-179)(35-104)
T0533	313	2	(7-89|181-296)(90-180)
T0543	887	4	(56-95)(96-140)(141-540)(541-884)
T0589	465	3	(24-188|271-369)(189-270)(370-464)
T0604	549	3	(11-94)(95-291|497-548)(292-496)
T0628	295	2	(6-132|279-295)(133-278)
T0629	216	2	(1-49|209-216)(50-208)
T0651	254	3	(1-95)(111-221)(222-254)
T0719	726	6	(39-133)(134-249)(257-356)(357-448)(449-563)(564-726)
T0739	770	4	(12-96)(97-154)(198-580)(581-769)

From CASP8 to CASP10, there are total 17 multi-domain targets with more than 3 segments, the minimum domain length >30, and only 2 segments in one domain.
